# Medical and Indirect Costs Associated with a Rocky Mountain Spotted Fever Epidemic in Arizona, 2002–2011

**DOI:** 10.4269/ajtmh.15-0104

**Published:** 2015-09-02

**Authors:** Naomi A. Drexler, Marc S. Traeger, Jennifer H. McQuiston, Velda Williams, Charlene Hamilton, Joanna J. Regan

**Affiliations:** Rickettsial Zoonoses Branch, Division of Vector-borne Diseases, National Center for Emerging and Zoonotic Infectious Diseases, Centers for Disease Control and Prevention, Atlanta, Georgia; Whiteriver Indian Health Service Hospital, Whiteriver, Arizona; Tribe A, Department of Health Services, Arizona; Tribe B, Department of Health Services, Arizona

## Abstract

Rocky Mountain spotted fever (RMSF) is an emerging public health issue on some American Indian reservations in Arizona. RMSF causes an acute febrile illness that, if untreated, can cause severe illness, permanent sequelae requiring lifelong medical support, and death. We describe costs associated with medical care, loss of productivity, and death among cases of RMSF on two American Indian reservations (estimated population 20,000) between 2002 and 2011. Acute medical costs totaled more than $1.3 million. This study further estimated $181,100 in acute productivity lost due to illness, and $11.6 million in lifetime productivity lost from premature death. Aggregate costs of RMSF cases in Arizona 2002–2011 amounted to $13.2 million. We believe this to be a significant underestimate of the cost of the epidemic, but it underlines the severity of the disease and need for a more comprehensive study.

Rocky Mountain spotted fever (RMSF) is a tick-borne rickettsial disease caused by the bacterium *Rickettsia rickettsii*. RMSF has been endemic in parts of the United States for well over a century, but emerged on tribal lands of Arizona in 2003.^1^ From 2000 to 2007, it was demonstrated that American Indians were experiencing a disproportionate burden of disease compared with other race groups (four times the burden of RMSF than whites).^2^

RMSF is known to cause an acute febrile illness potentially resulting in severe sequelae or death. Initial clinical presentation can vary widely and symptoms are often nonspecific in nature, making timely diagnosis difficult.^3^ When treatment is delayed past day 5 of symptoms, severe sequelae, such as neurological deficits or damage to internal organs, may occur.^4^ Such sequelae can cause irreparable damage requiring long-term hospital care and lifelong medical support. The case fatality rate of RMSF in Arizona is markedly higher than is observed elsewhere in the United States, averaging 7% of reported cases compared with < 1% nationally.^2^,^5^ Disease severity, long-term or permanent sequelae, and the potential for fatal outcome all contribute to the disease burden in this region. The costs of this epidemic are difficult to calculate, and must take into account not only acute care medical expenses but also potential loss of productivity due to illness or death.

A retrospective chart review was performed within two American Indian communities at the center of this epidemic. Medical charts for cases of RMSF diagnosed between June 1, 2002 and September 30, 2011 from Indian Health Service (IHS) facilities and referral hospitals were reviewed by Regan and others^4^ and Traeger and others.^3^ Case definitions used in this analysis follow those initiated by the original chart review.^3^,^4^ Table 1 gives selected characteristics of the case population from the analysis performed by Regan and others, as well as events at discharge.^4^

Costs associated with RMSF cases in these localities were considered for 2002–2011. To assess the financial burden of RMSF within affected communities, direct medical costs associated with acute medical care and indirect costs associated with productivity lost due to illness and premature death were calculated. All estimates were adjusted to 2011 dollar values based on inflation rates.^6^

The chart review captured visits to IHS and non-IHS facilities relating to acute disease management of diagnosed RMSF infections. We enumerated each visit type (classifying them as outpatient, emergency room (ER), inpatient/general admission, and inpatient/intensive care unit (ICU) admission). IHS reimbursement rates are standardized costs used by the Center for Medicaid Services to compensate IHS and tribal facilities for Medicaid reimbursable services. Rates are based on encounters, not specific services, and are considered “all-inclusive.” Daily rates were used for inpatient visits (including general hospital admission and ICU) and outpatient visits (including ER and clinic and hospital outpatient) were counted by encounter according to 2011 values.^7^ Inpatient days were calculated using dates of admission and discharge. The communities included in this study have limited on-site hospital facilities and treatment capacity for severe cases, and must transfer patients requiring intensive care. As such, cost of transfer was also included in the estimation of acute medical costs. Costs of air evacuations were negotiated locally and were estimated at $1,500 per one-way trip (Traeger, M. S., personal communication). Estimated value is consistent with air ambulance fee-for-service charges reported by the Arizona Health Care Cost Containment System (AHCCCS).^8^ For each patient, the cost per visit was multiplied by the number of each respective visit type and added to the cost of transfer, if applicable. The sum of these values is the estimate of acute medical costs associated with RMSF in these two communities.

Costs associated with acute productivity lost due to disease were based on estimates calculated by Grosse and others.^9^ The hourly rates of compensation used in the calculation of daily, annual, and lifetime production in the Grosse and others' paper were based on national averages from the Bureau of Labor and Statistics.^10^ National averages for annual income are nearly twice as large as annual income for the population in question, so a correction factor was applied to the Grosse estimates to compensate for this difference and more precisely address local rates.^10^ The correction factor used gender-specific average income of American Indians living in Arizona over gender-specific annual income for all ages provided in the Grosse paper.^9^,^10^ It was assumed, variations in compensation by age and time spent in market and household tasks did not differ between the study population and the national average.

The number of days of productivity lost due to RMSF was calculated by combining the number of days spent at each health-care visit (this would include 1 day for outpatient or ER visits and any inpatient days) plus 4 days for recovery regardless of disease severity. Age and gender-specific daily production values from Grosse and others,^9^ which were adjusted for inflation and local annual income, were then applied to the number of productivity days lost. For cases involving children (persons under 15 years of age) lost productivity for a 30-year-old female was used to serve as the productivity lost by a caregiver.

To calculate potential earnings lost from a premature death due to disease, we used the age- and gender-specific lifetime productivity estimates from Grosse and others, and adjusted them based on local annual income, as was done for acute productivity lost.^9^ Using the age at death for each of the fatal cases, we applied the population-adjusted age and gender-specific lifetime production lost at a 3% discount rate.

Two hundred and five cases were identified in the medical chart review according to the probable and confirmed case definitions. Twenty-nine people were admitted to the ICU and 15 people died. Table 2 shows the results for acute medical costs for all 205 cases. Summary of daily production value lost due to acute disease was $181,100 (95% confidence interval [CI]: $175,954, $186,240). On average, 1.5 (range 0–8) days were lost for outpatient visits (clinic and ER visits), 6.9 (range 0–55) days were lost for days spent in the hospital, and 4 days were added for the recovery of every patient. Lifetime productivity lost due to premature death from RMSF was estimated at $11,631,998 (95% CI: $11,304,814, $11,959,182). Aggregate economic costs of RMSF 2002–2011 surmounted to $13,184,968 (95% CI: $12,852,638, $13,517,292), as shown in Table 3.

The recent emergence of RMSF in Arizona has left devastation in its wake. This severe, but relatively isolated outbreak has cost millions of dollars in medical fees and economic productivity lost. Lifetime productivity loss accounts for 88% of the overall cost of disease in this evaluation. The average lifetime productivity lost per fatal case of RMSF ($775,467 per death) is far greater than similar estimates for pneumococcal disease ($140,862 per death) and West Nile Virus (WNV) ($293,960 per death).^11^,^12^ This high value, in comparison to other infectious diseases, may be attributed to the age of fatal cases in this review. The median age of death from RMSF in these two communities is 14 years (range 1–78), whereas the median age of fatal cases in WNV study was 76 years, and more than 80% of deaths relating to pneumococcal disease were among adults 65 years and older.^11^,^12^ This RMSF epidemic largely affects previously healthy children and young adults who would otherwise have the most potential to contribute economically to society.

Acute cost estimates provided in this study use all-inclusive rates reimbursed by Medicaid, rather than itemized billing, and do not represent the sum of actual expenses as direct billing information was not available. All-inclusive rates are not specific to the treatment of RMSF, and generally represent the minimum costs. Furthermore, expensive treatments, such as extracorporeal membrane oxygenation (ECMO), which cost upward of $16,000 for every 24 hours, were used to treat patients in our cohort but were not accounted for in all-inclusive rates.^13^ As the chart review was cross-sectional in nature, long-term costs, such as rehabilitation and ambulatory care, as well as loss of productivity due to disability, were not included and could significantly increase the estimated costs of disease. These calculations likely represent a gross underestimation of the costs sustained, but are valuable in that they underline the social and economic impact on affected communities and emphasize the need for a more comprehensive study to document the economic impact of this disease.

## Figures and Tables

**Table 1:** Selected characteristics of RMSF case population in Arizona, 2002–2011

Characteristic	Proportion of cases reporting
Male gender	106/205 (52%)
Median age (range)	11 years (7 months–78 years)
History of tick exposure	73/132 (55%)
Select severe sequelae	
Respiratory failure	22/204 (11%)
Disseminated intravascular coagulopathy	18/204 (9%)
Renal insufficiency	17/203 (8%)
Multisystem organ failure	14/204 (7%)
Acute respiratory distress syndrome	13/204 (6%)
Coma	11/203 (5%)
Digital necrosis	3/201 (2%)
Events at discharge	
Long-term sequelae	5/187 (3%)
Rehab facility	2/188 (1%)
Specialty follow-up	5/186 (3%)
Fatal outcome	15/205 (7%)

RMSF = Rocky Mountain spotted fever.

**Table 2:** Acute medical costs based on IHS all-inclusive reimbursement rates from Medicaid

Item	Number of people reporting at least one	Total number of encounters or days	Cost per unit ($)	Total cost ($)
ER visits	170	256	294	75,264
Outpatient or clinic visits*	81	125	294	36,750
Inpatient days†	82	360	2,034	732,240
Transfers	48	48	1,500	72,000
ICU days	29	224	2,034	455,616
				Bulk total
				1,371,870

ER = emergency room; ICU = intensive care unit; IHS = Indian Health Service.

* Visit not resulting in an admission, excluding visits to ER.

† Excludes days spent in the ICU.

**Table 3:** Summary of direct and indirect costs associated with RMSF in Arizona, 2002–2011

	Point estimate	Lower bound	Upper bound
Direct costs			
Acute medical costs	$1,371,870	$1,371,870	$1,371,870
Long-term medical costs	NA	–	–
Indirect costs			
Acute loss to productivity	$181,100	$175,954	$186,240
Long-term loss of productivity due to disability	NA	–	–
Lifetime lost due to death	$11,631,998	$11,304,814	$11,959,182
Total	$13,184,968	$12,852,638	$13,517,292

NA = not addressed in this study due to unavailability of relevant clinical information; RMSF = Rocky Mountain spotted fever.
